# High Prevalence of Anti-HCV Antibodies in Two Metropolitan Emergency Departments in Germany: A Prospective Screening Analysis of 28,809 Patients

**DOI:** 10.1371/journal.pone.0041206

**Published:** 2012-07-25

**Authors:** Johannes Vermehren, Beate Schlosser, Diana Domke, Sandra Elanjimattom, Christian Müller, Gudrun Hintereder, Karin Hensel-Wiegel, Rudolf Tauber, Annemarie Berger, Norbert Haas, Felix Walcher, Martin Möckel, Ralf Lehmann, Stefan Zeuzem, Christoph Sarrazin, Thomas Berg

**Affiliations:** 1 Medizinische Klinik 1, Klinikum der J. W. Goethe-Universität, Frankfurt am Main, Germany; 2 Medizinische Klinik m.S. Hepatologie und Gastroenterologie, Charité – Universitätsmedizin, Berlin, Germany; 3 Institut für Laboratoriumsmedizin, Klinische Chemie und Pathobiochemie, Charité – Universitätsmedizin, Berlin, Germany; 4 Zentrallabor, Klinikum der J. W. Goethe-Universität, Frankfurt am Main, Germany; 5 Institut für Medizinische Virologie, Klinikum der J. W. Goethe-Universität, Frankfurt am Main, Germany; 6 Klinik für Unfall- und Wiederherstellungschirurgie, Charité – Universitätsmedizin, Berlin, Germany; 7 Klinik für Unfall-, Hand- und Wiederherstellungschirurgie, Klinikum der J. W. Goethe-Universität, Frankfurt am Main, Germany; 8 Medizinische Klinik m.S. Kardiologie und Arbeitsbereich Notfallmedizin/Rettungsstellen, Nord-Campi, Charité – Universitätsmedizin, Berlin, Germany; 9 Medizinische Klinik 3, Klinikum der J. W. Goethe-Universität, Frankfurt am Main, Germany; 10 Klinik und Poliklinik für Gastroenterologie und Rheumatologie, Sektion Hepatologie, Universitätsklinikum Leipzig, Germany; University of Modena & Reggio Emilia, Italy

## Abstract

**Background and Aims:**

The prevalence of hepatitis C virus (HCV) antibodies in Germany has been estimated to be in the range of 0.4–0.63%. Screening for HCV is recommended in patients with elevated ALT levels or significant risk factors for HCV transmission only. However, 15–30% of patients report no risk factors and ALT levels can be normal in up to 20–30% of patients with chronic HCV infection. The aim of this study was to assess the HCV seroprevalence in patients visiting two tertiary care emergency departments in Berlin and Frankfurt, respectively.

**Methods:**

Between May 2008 and March 2010, a total of 28,809 consecutive patients were screened for the presence of anti-HCV antibodies. Anti-HCV positive sera were subsequently tested for HCV-RNA.

**Results:**

The overall HCV seroprevalence was 2.6% (95% CI: 2.4–2.8; 2.4% in Berlin and 3.5% in Frankfurt). HCV-RNA was detectable in 68% of anti-HCV positive cases. Thus, the prevalence of chronic HCV infection in the overall study population was 1.6% (95% CI 1.5–1.8). The most commonly reported risk factor was former/current injection drug use (IDU; 31.2%) and those with IDU as the main risk factor were significantly younger than patients without IDU (p<0.001) and the male-to-female ratio was 72% (121 vs. 46 patients; p<0.001). Finally, 18.8% of contacted HCV-RNA positive patients had not been diagnosed previously.

**Conclusions:**

The HCV seroprevalence was more than four times higher compared to current estimates and almost one fifth of contacted HCV-RNA positive patients had not been diagnosed previously.

## Introduction

Chronic infection with the hepatitis C virus (HCV) affects an estimated 2–3% of the world's population and is a leading cause of cirrhosis and hepatocellular carcinoma [1]. With the former standard of care, a combination of pegylated interferon-alfa plus ribavirin, the virus could be permanently eradicated in 42–46% of patients with HCV genotype 1 only [2], [3]. However, with the addition of recently approved direct-acting HCV protease inhibitors, treatment success rates have been substantially improved by approximately 20–40% [4], [5], [6], [7].

Patients with advanced liver fibrosis or cirrhosis are less likely to be cured compared to those without relevant fibrosis [8]. Moreover, chronic HCV infection is asymptomatic in the majority of patients and diagnosis is therefore often delayed until more advanced stages of fibrosis are present. Thus, early diagnosis is desirable to optimize overall treatment success. Despite this, national screening programs and surveillance systems are largely heterogeneous across Europe, with reported HCV antibody prevalences ranging from 0.4% to 3.5% by country and from 0.2% to 10.4% by region within countries [9]. Differences in prevalence are largely attributed to variances in transmission routes and differences in public health policies [10].

In Germany, the HCV seroprevalence has been estimated to be in the range of 0.4–0.63% in the general population according to two community-based studies conducted in 1993 to 1996 and 1998, respectively [11], [12], [13]. In clinical practice, HCV antibody testing is routinely performed in risk populations only (e.g. blood donors, injecting drug users etc.) and in those with unexplained liver enzyme elevations [14]. However, it has been reported that an estimated 15–30% of patients with chronic HCV do not report any risk factors [15] and approximately 20–30% are reported to have persistently normal liver enzymes [16], [17], [18]. Therefore, the number of unreported cases, especially in metropolitan areas with many high-risk groups, may be much higher than previously assumed.

The aim of this study was to assess the prevalence of anti-HCV antibodies, HCV-RNA and associated risk factors in patients visiting emergency departments of two urban, tertiary care hospitals.

## Materials and Methods

### Study population

The study was conducted in compliance with the declaration of Helsinki and approval was obtained from the Ethics Committee of the Charité – Universitätsmedizin, Berlin, Germany and the Ethics Committee of the Medical Faculty of the J. W. Goethe University, Frankfurt, Germany. In accordance with the Ethics Committees requirements at the two participating study sites, patients were informed of the study procedure and a notice was displayed on a board.

Between May 2008 and October 2009, excess serum was retained from all consecutive patients of 18 years of age or older who presented to the internal medicine and traumatology emergency departments at the Charité, Campus Virchow-Klinikum in Berlin and who had a blood sample taken as part of their routine diagnostic work up. In addition, excess serum was also retained from consecutive patients of 18 years of age or older who presented to the J. W. Goethe University Hospital emergency department in Frankfurt between September 2009 and March 2010.

Demographic data, including age, gender, ethnicity and routine laboratory values, including serum aminotransferase levels were collected from hospital admission charts, where available. Upper limit of normal (ULN) values for alanine aminotransferase and aspartate aminotransferase levels were defined as 35 U/mL in females and 50 U/mL in males.

### HCV antibody screening and sample interpretation

HCV antibody screening of blood samples was performed on the same day they were drawn using a fully automated electrochemiluminescence immunoassay (Elecsys**®** Anti-HCV immunoassay; Roche Diagnostics, Penzberg, Germany) on a cobas e 601 platform. Serum samples with a signal/cut-off (s/co) ratio <0.9 were considered non-reactive, those with a s/co ratio ≥1.0 were considered reactive, and samples with results between 0.9 and <1.0 were interpreted as indeterminate.

All samples tested positive or indeterminate by the Elecsys Anti-HCV immunoassay were subsequently stored at −20° to −25°C and retested with the ARCHITECT anti-HCV test (Abbott Diagnostics, Wiesbaden, Germany), a fully automated chemiluminescence micro particle immunoassay within 1–5 days. ARCHITECT results were interpreted as non-reactive (s/co <1) or reactive (s/co ≥1).

Samples with reactive results according to both assays were considered anti-HCV positive, samples that were interpreted as reactive by the Elecsys assay but non-reactive by the ARCHITECT assay were counted as inconsistent results (IR).

### HCV-RNA testing and genotyping

Samples with reactive anti-HCV results according to both assays were further tested for the presence of HCV-RNA using the real-time PCR-based COBAS Ampliprep/COBAS TaqMan HCV-RNA assay (Roche Diagnostics, Penzberg, Germany; lower limit of quantitation, 15 IU/mL), if sufficient stored (−20 to −25° C) left-over serum was available. IR samples were also tested for HCV-RNA, depending on availability of leftover serum. Samples with detectable but non-quantifiable HCV-RNA results (i.e. <15 IU/mL) were considered HCV-RNA positive.

In Berlin HCV genotyping was performed on samples with sufficient leftover volume and viral load (>1000 IU/mL) using the VERSANT HCV Genotype 2.0 line probe assay (LiPA; Siemens Healthcare Diagnostics, Eschborn, Germany). In Frankfurt known genotypes were recorded from patient charts. Genotyping on these patients had also been performed with the VERSANT HCV Genotype 2.0 line probe assay.

All assays used in this study were performed and interpreted according to the manufacturer's instructions.

### Patient contacting, risk factors and HCV awareness

Patients with reactive anti-HCV antibody results were contacted by telephone and/or mail and appropriate diagnostic and therapeutic follow-up was offered. In addition, knowledge of HCV antibody status, presence of risk factors, disease history and past antiviral therapies were recorded at the Berlin study site. Furthermore, the presence of risk factors for possible HCV infection was also recorded in a random sample of 391 anti-HCV negative patients who served as controls and risk factors were subjected to logistic regression analysis based on 936 patients (anti-HCV positive in Berlin, n = 535; anti-HCV negative, n = 391). Age and gender distribution in the control group was similar to that in the overall anti-HCV negative population.

At the Frankfurt study site, only knowledge of HCV antibody status was recorded.

In Germany, newly diagnosed patients with HCV infection have to be reported to local health authorities and, ultimately, to the Robert Koch Institute, a federal institution for disease control and prevention. Therefore, all newly diagnosed patients were also notified by the local health authorities to ensure appropriate follow-up.

### Statistical analyses

Clinical and laboratory characteristics of patients were expressed as mean ± standard deviation or median and range, as appropriate. Unadjusted prevalence was calculated and 95% confidence intervals were based on a binominal distribution.

Comparisons were made using the chi-square test or the Fisher's exact test for categorical variables and Mann-Whitney U-test for continuous variables, as appropriate. Correlations between variables were performed using Spearman's correlation coefficients. To test for associations between risk factors for HCV infection and HCV seropositivity, univariate and multivariate models were employed. For all tests, a *p*-value of less than 0.05 was judged to be statistically significant.

All statistical analyses were performed using BiAS for Windows, version 9.08 (epsilon 2010, Frankfurt, Germany) or IBM SPSS Statistics for Windows, version 19.0 (SPSS/IBM, Somers, NY, USA).

## Results

### Anti-HCV antibody prevalence and patient characteristics

A total of 28,809 patients (52% males, mean age, 51.9±20 years) were screened for the presence of anti-HCV antibodies at the two participating study sites. Of these, 1,060 patients (3.7%; 95% CI: 3.5–3.9) were tested positive for the presence of anti-HCV antibodies with the Elecsys Anti-HCV assay. Subsequent retesting of anti-HCV antibody positive and indeterminate patient samples with the ARCHITECT anti-HCV assay confirmed the presence of anti-HCV antibodies in 757/1,060 (71.4%) cases. Thus, an overall prevalence of 2.6% (95% CI: 2.4–2.8) was recorded, 2.4% (95% CI: 2.2–2.6; n = 535/22,490) at the Berlin study site and 3.5% (95% CI: 3.1–4.0; n = 222/6,319) in Frankfurt. Screening results according to the different study sites and distribution among internal medicine and traumatology emergency departments are shown in Table 1. Whereas significantly more anti-HCV positive patients were visiting the internal medicine wards (2.7% vs. 2.4%; p<0.001), the number of HCV-RNA positive patients was higher among those visiting the trauma departments (71% vs. 67%; p<0.001).

**Table 1:** Distribution of anti-HCV and HCV-RNA status for each of the two study sites alone and all patients combined.

Study site	all patients	Berlin	Frankfurt
Number of patients screened	28,809	22,490	6,319
HCV status, n (%)			
anti-HCV+	757 (2.6)	535 (2.4)	222 (3.5)
HCV-RNA+ (% of tested)	465/685 (68)	346/503 (69)	119/182 (65)
HCV-RNA− (% of tested)	220/685 (32)	157/503 (31)	63/182 (35)
Internal ER, n (%)	20,642 (72)	17,024 (76)	3,618 (57)
anti-HCV+	562 (2.7)	399 (2.3)	163 (4.5)
HCV-RNA+ (% of tested)	337/504 (67)	253/373 (68)	84/131 (64)
HCV-RNA− (% of tested)	167/504 (33)	120/373 (32)	47/131 (36)
Trauma ER, n (%)	8,167 (28)	5,466 (24)	2,701 (43)
anti-HCV+	195 (2.4)	136 (2.5)	59 (2.2)
HCV-RNA+ (% of tested)	128/181 (71)	93/130 (72)	35/51 (69)
HCV-RNA− (% of tested)	53/181 (29)	37/130 (28)	16/51 (31)

Serum samples for HCV-RNA analysis were available from 685 out of 757 anti-HCV positive patients.

The highest anti-HCV prevalence was found in patients aged 40–59 (4.1%). Thirty-eight and 24 patients, respectively, needed to be screened to identify one anti-HCV positive patient in the overall study population and in patients aged 40–59 only. Among anti-HCV positive patients, males were significantly younger than females (p<0.001) and peaks were observed in males aged 40–59 years and in females aged ≥60 years. The majority of anti-HCV positive patients were of German origin (67.8%) and Eastern Europeans made up the largest group among immigrants (11.2%; Table 2).

**Table 2:** Patient characteristics.

Screening site	All patients (n = 28,809)				Berlin (n = 22,490)			Frankfurt (n = 6,319)		
	anti-HCV+ (n = 757)	anti-HCV+ and		*p*-value	anti-HCV+ (n = 535)	anti-HCV+ and		anti-HCV+ (n = 222)	anti-HCV+ and	
		HCV-RNA+ (n = 465)	HCV-RNA− (n = 220)			HCV-RNA+ (n = 346)	HCV-RNA− (n = 157)		HCV-RNA+ (n = 119)	HCV-RNA− (n = 63)
Mean age in years ± SD	52.9±16.8	54.5±16.7	50.4±15.7	0.002	54±16.7	54±16.9	52 ±15.6	51±16.7	55±16.1	48±15.8
Male, n (%)	456 (60.2)	286 (74.1)	128 (58.2)	n.s.	312 (58.3)	212 (61.3)	85 (54.1)	144 (64.9)	74 (62.2)	43 (68.3)
HCV genotype, n (% of known)										
1a/b		210 (65.6)				176 (64.9)			34 (69.4)	
2		17 (5.3)				16 (5.9)			1 (2.0)	
3		61 (19.1)				49 (18.2)			12 (24.5)	
4		17 (5.3)				15 (5.5)			2 (4.1)	
Other		15 (4.7)				15 (5.5)			-	
Unknown		145				75			70	
ALT and/or AST tested										
n, (%)	656 (86.7)	408 (87.7)	189 (85.9)	n.s.	457 (85.4)	298 (86.1)	134 (85.4)	199 (89.6)	110 (92.4)	55 (87.3)
>ULN^a^, n (%)	341 (52.0)	249 (61.0)	61 (32.3)	<0.001	232 (50.8)	180 (60.4)	44 (32.8)	109 (54.8)	69 (62.7)	17 (30.9)
Ethnicity, n (%)										
German	513 (67.8)	316 (68.0)	142 (64.5)	n.s.	370 (69.2)	241 (69.7)	101 (64.3)	143 (64.4)	75 (63.0)	41 (65.1)
Eastern European	85 (11.2)	55 (11.8)	24 (10.9)	n.s.	53 (9.8)	37 (10.7)	16 (10.3)	32 (14.4)	18 (15.1)	8 (12.7)
Turkish	42 (5.5)	27 (5.8)	12 (5.5)	n.s.	32 (6.0)	22 (6.3)	9 (5.7)	10 (4.5)	5 (4.2)	3 (4.8)
Other/unknown	117 (15.5)	67 (14.4)	42 (19.1)	n.s.	80 (15.0)	46 (13.3)	31 (19.7)	37 (16.7)	21 (17.7)	11 (17.4)
Knowledge of HCV status, n (% of patients with available data)										
Yes	492 (77.8)	323 (81.2)	131 (72.2)	0.0096	340 (73.8)	237 (78.5)	89 (65.4)	152 (88.9)	86 (89.6)	42 (87.5)
No	140 (22.2)	75 (18.8)	53 (28.8)		121 (26.2)	65 (21.5)	47 (34.6)	19 (11.1)	10 (10.4)	6 (12.5)
No data	125	67	36		74	44	21	51	23	15
No. of patients with past antiviral therapy, n (%)	104 (13.7)	59 (12.7)	42 (19.1)	0.0373	104 (19.4)	59 (17.1)	42 (26.8)	no data		
No. of patients with SVR, n (%)			39^b^ (17.7)				39^b^ (24.8)	no data		

a ULN = upper limit of normal; ^b^of 42 HCV-RNA negative patients, 3 were still on antiviral treatment at the time of study inclusion.

Of all HCV seropositive patients, 65% knew that they were or had been infected. Knowledge of HCV status was significantly higher in those with confirmed chronic HCV infection compared to those who were seropositive only (p = 0.0096) and significantly more patients with chronic HCV infection from Frankfurt were aware of their status compared to patients from Berlin (p = 0.0161). Patient characteristics according to serostatus and nucleic acid analysis are presented in Table 2.

### HCV-RNA analysis and genotype distribution

Among the 757 patient samples with positive anti-HCV results, 685 (90.5%) were available for HCV-RNA testing. In addition, HCV-RNA testing was also performed in 85 patients with inconsistent serologic results (Elecsys positive/ARCHITECT negative).

HCV-RNA was detectable in 465/685 (67.9%) anti-HCV positive patients (Table 1). Thus, the prevalence of chronic hepatitis C infection in the overall study population was 1.6% (95% CI 1.5–1.8; n = 465/28,809). Among HCV-RNA positive patients, 41 (Berlin, n = 40; Frankfurt, n = 1) had detectable viral load levels below the assay's limit of quantitation (i.e. <15 IU/mL). Among the 85 patients with inconsistent serologic results, 5 had positive HCV-RNA results <15 IU/mL whereas the remaining patients were HCV-RNA negative.

Forty-two patients (26.8%) from the Berlin study site who tested positive for anti-HCV antibodies but negative for HCV-RNA reported a history of antiviral treatment. The remaining 115 (73.2%) patients had either cleared HCV-RNA spontaneously or they had false positive anti-HCV results. However, as all 115 patients had concordant positive anti-HCV results according to both assays, spontaneous clearance may have been more likely.

HCV genotype 1 was the most prevalent genotype (65.6%, n = 210) in HCV-RNA positive patients, followed by genotype 3 (19.1%; n = 61) and 2 (5.3%; n = 17).

### Liver enzymes

Alanine aminotransferase (ALT) and/or aspartate aminotransferase (AST) levels were determined in 656 (87%) of anti-HCV antibody positive patients. Elevated ALT and/or AST levels were present in 52% of patients only. However, in patients with detectable HCV-RNA, 61% had elevated ALT/AST levels and this was significantly more compared to those with anti-HCV only (32%; p<0.001). However, elevated ALT/AST levels were poorly correlated with chronic HCV infection (r = 0.27, p<0.001). When specifically looking at patients with negative HCV-RNA, there was no difference in BMI, alcohol or nicotine use between patients with or without ALT/AST elevation (data not shown).

### Risk factors

Typical risk factors for HCV transmission were recorded in 319/535 (60%) patients at the Berlin screening site (Table 3). Current or former IDU was reported as the primary risk factor for HCV infection in 167 patients (31%). Other risk factors included a surgical procedure before 1992 (when commercial anti-HCV serological testing became available; 19%), past history of blood transfusion before 1992 (9%), past history of solid organ transplantation (8%), hemodialysis (4%) and coagulation disorders (2%).

**Table 3:** Self-reported risk factors for HCV transmission in the anti-HCV positive population at the Berlin study site.

Risk factor, n (%)	
Any risk factor	319 (59.6)
IVDU	167 (31.2)
Surgical procedure before 1992	103 (19.3)
Transfusion of blood products before 1992	48 (9.0)
Solid organ transplantation	45 (8.4)
Haemodialysis	20 (3.7)
Health care worker	16 (2.2)
Sexual contact with HCV infected person	8 (1.5)
Coagulation disorder	7 (1.3)
Prison inmate	2 (0.4)

IVDU, intravenous drug-use.

Multiple responses were possible. Percentages are given in relation to all anti-HCV positive patients (n = 535).

Univariate analysis showed that current or former IDU, surgical procedures before 1992, past history of blood transfusion before 1992 and elevated liver enzymes were all associated with anti-HCV seropositivity. Risk factors that remained independently associated with anti-HCV seropositivity in the multivariate analysis included past history of blood transfusion before 1992 (OR = 15.5, 95% CI: 4.7–51.7, p<0.001) and elevated liver enzymes (OR = 3.0 1.3–7–4, p = 0.02).

Patients with IDU as the main risk factor were significantly younger than those without IDU (p<0.001) and the male-to-female ratio was 72% (121 vs. 46 patients; p<0.001). Furthermore, patients with IDU had been diagnosed more often prior to the current screening program (90%), while those without IDU had not been diagnosed in 41% of cases.

### Eligibility for antiviral treatment

**Figure 1:**
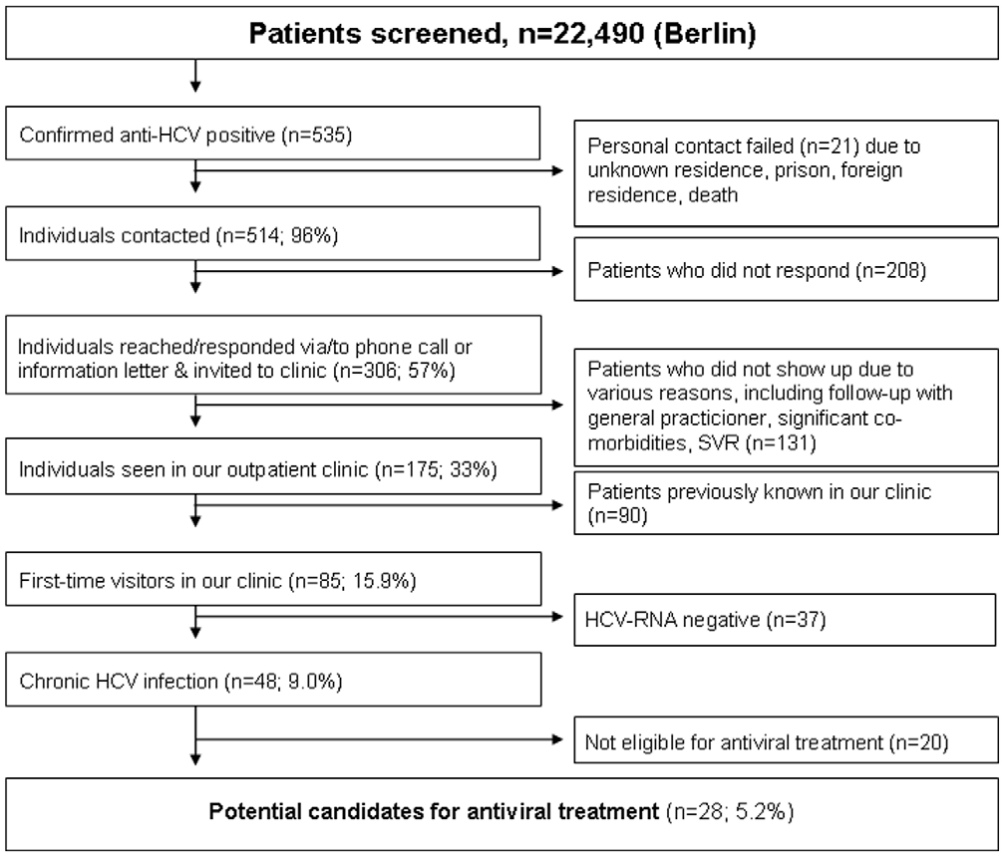
Study flow-chart showing outcome of the Berlin screening population with respect to eligibility for antiviral treatment. Percentages are given in relation to anti-HCV positive patients (n = 535).

Five hundred and fourteen out of 535 HCV seropositive patients (96%) from Berlin were contacted by phone or in writing. Of these, 175 (33%) followed our invitation to get expert advice in our hepatology outpatient clinic. Among patients not previously seen in our clinic, 48 had confirmed chronic HCV infection and 28 (5%) were considered eligible for antiviral treatment while 20 had medical conditions precluding them from pegylated interferon/ribavirin therapy (e.g. drinking problem, active IDU, moderate-to-severe depression; Figure 1).

## Discussion

Comprehensive epidemiological data on HCV prevalence and associated risk factors are limited. Recent findings suggest that HCV seroprevalence rates in Germany vary significantly by region, with higher prevalence rates in Western Germany compared to Eastern Germany and in urban versus rural areas [19], [20]. These regional variations may have direct implications for federal and local public health measures regarding prevention and control of HCV infection.

To our knowledge, our study represents the largest prospective data analysis on HCV prevalence in patients admitted to emergency departments worldwide.

We applied a two-step screening approach, using the Elecsys Anti-HCV assay as the primary screening test followed by confirmatory testing with the Architect Anti-HCV assay in all Elecsys-positive and indeterminate samples. An assay comparison study was not intended. However, with the Architect serving as the reference assay, Elecsys Anti-HCV had a specificity of 99% at the two participating study sites as was also observed in a recent assay validation study [21]. Interestingly, all specimens with inconsistent anti-HCV results (Elecsys positive/ Architect negative) that were later tested for the presence of HCV-RNA (n = 85), yielded negative or <15 IU/mL PCR results. It must be noted, however, that previous studies had not shown lower sensitivities for the Elecsys assay in comparison to competitor assays [21], [22] and our results should be interpreted with caution as no head-to-head assay comparison was performed.

The main finding of our study was that the HCV seroprevalence was 2.6% (95% CI: 2.4–2.8) at our two institutions combined, which is more than four times higher than the estimated prevalence in the general German population [11], [12]. The prevalence was higher in Frankfurt (3.5%) compared to Berlin (2.4%). The overall high HCV prevalence may be partly explained by the urban study setting as well as the fact that high-risk populations (e.g., injecting drug users, homeless people and other patients not enrolled in regular health plans) were not excluded from our study. Other risk groups (e.g., patients with coagulation disorders or liver transplant candidates) may even have been overrepresented as they are regular visitors to tertiary care university hospitals and thus may account for selection bias.

In the past, HCV seroprevalence estimates have been performed in different populations and regions but usually involved considerably lower sample sizes [10], [23], [24]. In a study comprising 2,523 patients who presented to the emergency department at a university hospital in Baltimore, USA, in 1988, the HCV seroprevalence was found to be 18% with a particularly high prevalence among minorities [23]. However, nucleic acid testing was not available at that time and antiviral treatment was still in the early stages of development [25]. In a more recent study from Switzerland [24], the anti-HCV prevalence among 5,036 patients who were admitted to the emergency department of a university hospital in Berne was calculated to be 2.7%, and this is in line with our own findings. However, in that study, HCV-RNA was tested in 15.6% of anti-HCV positive patients only and thus, little information on persisting HCV infection was available.

Another important finding of our study is that the proportion of patients with detectable HCV-RNA was 68%, which is lower than that found in the German National Health Survey (84%) conducted in 1998 [12]. Interestingly, a similar decline was recently reported from France where the proportion of HCV-RNA positivity among anti-HCV positive adults aged 20–59 years declined from 81% in 1994 to 57% in 2004 and this was most likely attributable to an increased treatment activity [26]. Although increased treatment activity has also been reported from Germany, our own data may not be directly comparable with those obtained in the French population-based study.

Analysis of genotype distribution in HCV-RNA positive patients yielded similar results to those of a recent comprehensive analysis in 9,455 patients from different parts of Germany [19]. That is, genotype 1 was the type most commonly found in our study population (66%), followed by genotype 3 (19%) and 2 (5%). However, in line with a shift in infection sources, decreasing numbers of HCV genotype 1b (the genotype most commonly associated with transmission via blood transfusions) infections and increasing prevalence of HCV genotype 3 (the genotype most commonly associated with IDU) have been reported [19].

In line with previous findings [19], [27], [28], the anti-HCV prevalence was higher in males than in females at both study sites, and males were younger with the highest prevalence among those aged 40–59 years (4.1%). This may be explained by gender-specific risk behavior, as IDU and, more recently, sexual transmission have been found to be more frequent in men whereas the majority of women may have been infected via transfusion of blood products or anti-D prophylaxis by immunoglobulin prior to the commercial introduction of anti-HCV screening tests in the early 1990s [29], [30], [31], [32]. Interestingly, the two aforementioned German community-based studies found a higher, albeit not significant HCV seroprevalence in females, indicating that IDUs may have been underrepresented in these studies.

In our study, patients with a migration background from Eastern Europe made up the largest group of non-German anti-HCV carriers and this is in line with previous findings [19]. Recent population based anti-HCV prevalence estimates from former Communist countries were up to 8–9 times higher compared to Germany, ranging from 1.5–2% in Poland and the Czech Republic to 3.5% in Romania. The main reasons for these high prevalence rates are believed to be infections due to blood transfusions before 1995 and incomplete sterilized medical equipment, which continues to be a source in rural areas [10]. The high proportion of anti-HCV positive patients from Eastern Europe in our study most likely reflects the significant increase in immigration from former Communist countries since 1990.

In a recent study, targeted age-based screening was suggested based on a mathematical approach using a birth cohort of United States residents born between 1946 and 1970 [33]. In their model, McGarry and co-workers predicted a significant health benefit and improved cost-effectiveness over risk-based screening programs that are currently in use. Our own prevalence data may support such an approach.

Contrary to previous findings, knowledge of HCV status was high (78%) among anti-HCV positive patients and this may again reflect the affiliation with university hepatology outpatient clinics.

In our study, almost one third of anti-HCV positive patients who were contacted regarding risk behavior reported former or current IDU. Indeed, IDU is currently believed to be the major risk factor in most Western European countries where up to 90% of HCV-infected patients have reported IDU as the primary transmission factor [10]. Interestingly, the vast majority (90%) of patients with a history of IDU had been diagnosed prior to our study, indicating that effective screening measures are in place in this particular patient population. In contrast, 41% of patients who did not report IDU as a risk factor were unaware of their HCV serostatus.

A further important finding of our study is that 39% of patients with detectable HCV-RNA had normal serum aminotransferase levels. This constitutes a much higher percentage than previously reported [18], [19] and may be of immediate clinical significance as it supports the applicability of emergency departments as targets for an increased screening activity, especially when taking into account that one half of contacted anti-HCV positive patients did not report any risk factors for HCV infection in our study.

Eligibility for treatment was only assessed in the Berlin patient cohort. Here, out of 346 patients with chronic HCV infection, 28 (8%) were eligible for immediate antiviral therapy. This low number may be explained by restrictive eligibility criteria and only 33% of anti-HCV positive patients were assessed in our hepatology outpatient clinic whereas the majority of contacted patients preferred to be further evaluated by their primary care physicians. Thus, these data should be interpreted with caution.

Our study has several limitations: First, due to the study setting and design it cannot be extrapolated to the general German population. Second, given the fact that not all patients who presented to our emergency departments had a blood sample taken, a possible recruitment bias needs to be taken into account. Nevertheless, our data represent one of the largest cross-sectional patient samples ever to be screened. Finally, the lack of a structured patient questionnaire weakens our data on risk behavior, knowledge of HCV serostatus and treatment eligibility.

In conclusion, our data provide evidence of a high HCV seroprevalence among urban emergency department patients and clearly support the importance of re-defining risk groups and designing screening programs accordingly (e.g. birth-cohort screening). In light of recently improved cure rates for chronic HCV, expanding access to treatment should be encouraged.
